# 醛酮化合物色谱保留指数的集成全息定量构效关系模型

**DOI:** 10.3724/SP.J.1123.2020.06011

**Published:** 2021-03-08

**Authors:** Bin LEI, Yunlei ZANG, Zhiwei XUE, Yiqing GE, Wei LI, Qian ZHAI, Long JIAO

**Affiliations:** 1.西安石油大学化学化工学院, 陕西 西安 710065; 1. College of Chemistry and Chemical Engineering, Xi’an Shiyou University, Xi’an 710065, China; 2.核工业二〇三研究所, 陕西 咸阳 712000; 2. No. 203 Research Institute of Nuclear Industry, Xianyang 712000, China; 3.庆安集团有限公司, 陕西 西安 710077; 3. Qing’an Group Co., Ltd., Xi’an 710077, China

**Keywords:** 集成建模, 全息定量构效关系, 醛酮化合物, 色谱保留指数, ensemble modeling, hologram quantitative structure-activity relationship, aldehydes and ketones, chromatographic retention index

## Abstract

色谱保留指数(retention index, RI)是色谱分析中的重要参数,不同化合物在不同极性固定相上具有不同的保留行为。醛酮化合物种类众多,实验测定其RI值的时间和经济成本高。该论文采用集成建模(ensemble modeling)结合全息定量构效关系(HQSAR)方法研究了醛酮化合物在2种固定相(DB-210和HP-Innowax)上色谱保留指数的定量构效关系(QSAR)模型。用外部测试集验证法和留一交叉验证法评估了所建立模型的预测能力。首先建立了34种被研究化合物的个体HQSAR模型。在固定相DB-210上,片段特性(FD)为“供体/受体原子(DA)”且片段尺寸(FS)为1~9时可得到最优个体模型,在固定相HP-Innowax上,FD为“DA”且FS为4~7时可得到最优个体模型,这两个模型的交叉验证相关系数( $q_{\mathrm{cv}}^{2}$ )分别为0.935和0.909,外部验证相关系数( $q_{\mathrm{ext}}^{2}$ )分别为0.925和0.927,一致性相关系数(CCC)分别为0.953和0.960,预测平方相关系数F2( $Q_{F2}^{2}$ )分别为0.922和0.918,预测平方相关系数F3( $Q_{F3}^{2}$ )分别为0.931和0.927。研究结果表明醛酮化合物的分子结构与RI值之间存在定量关系,用HQSAR方法可以建立二者之间的QSAR模型。其次,以4个预测准确度最高的个体HQSAR模型作为子模型通过算术平均建立了集成HQSAR模型。建立的集成HQSAR模型预测被研究化合物在DB-210和HP-Innowax固定相上RI值的 $q_{\mathrm{cv}}^{2}$ 分别为0.927和0.919, $q_{\mathrm{ext}}^{2}$ 分别为0.929和0.963, CCC分别为0.956和0.979, $Q_{F2}^{2}$ 分别为0.927和0.958, $Q_{F3}^{2}$ 分别为0.935和0.963。与个体HQSAR模型相比,建立的集成HQSAR模型预测准确度更高。这说明集成建模是提高HQSAR模型预测能力的有效方法,HQSAR与集成建模方法相结合可以用于研究和预测醛酮化合物的RI值。

## Abstract (en)

Chromatographic retention index (RI) is an important parameter for describing the retention behavior of substances in chromatographic analysis. Experimentally determining the RI values of different aldehyde and ketone compounds in all kinds of polar stationary phases is expensive and time consuming. Quantitative structure activity relationship (QSAR) is an important chemometric technique that has been widely used to correlate the properties of chemicals to their molecular structures. Irrespective of whether the properties of a molecule have been experimentally determined, they can be calculated using QSAR models. It is therefore necessary and advisable to establish the QSAR model for predicting the RI value of aldehydes and ketones. Hologram QSAR (HQSAR) is a highly efficient QSAR approach that can easily generate QSAR models with good statistics and high prediction accuracy. A specific fragment of fingerprint, known as a molecular hologram, is proposed in the HQSAR approach and used as a structural descriptor to build the proposed QSAR model. In general, individual HQSAR models are built in QSAR researches. However, individual QSAR models are usually affected by underfitting and overfitting. The ensemble modeling method, which integrate several individual models through certain consensus strategies, can overcome the shortcomings of individual models. It is worth studying whether ensemble modeling can improve the prediction ability of the HQSAR method in order to build more accurate and reliable QSAR models.

Therefore, this study investigates the QSAR model for chromatographic RI of aldehydes and ketones using ensemble modeling and the HQSAR method. Two individual HQSAR models comprising 34 compounds in two stationary phases, DB-210 and HP-Innowax, were established. The prediction ability of the two established models was assessed by external test set validation and leave-one-out cross validation (LOO-CV). The investigated 34 compounds were randomly assigned into two groups. Group Ⅰ comprised 26 compounds, and Group Ⅱ comprised 8 compounds. In the validation of the external test set, Group Ⅰ was employed to manually optimize the two fragment parameters (fragment distinction (FD) and fragment size (FS)) and build the HQSAR models. Group Ⅱ was used as the test set to assess the predictive performance of the developed models. For the DB-210 stationary phase, the optimal individual HQSAR model was obtained while setting the FD and FS to “donor/acceptor atoms (DA)” and 1-9, respectively. For the HP-Innowax stationary phase, the optimal individual HQSAR model was obtained by setting the FD and FS to “DA” and 4-7 respectively. The squared correlation coefficient of cross validation ( $q_{\mathrm{cv}}^{2}$ ), concordance correlation coefficient (CCC), squared correlation coefficient of external validation ( $q_{\mathrm{ext}}^{2}$ ), predictive squared correlation coefficient ( $Q_{F2}^{2}$ and $Q_{F3}^{2}$ ) of the two models for predicting the RI value were 0.935 and 0.909, 0.953 and 0.960, 0.925 and 0.927, 0.922 and 0.918, and 0.931 and 0.927, respectively. The results of the two validations show that there is a quantitative relationship between the molecular structure of these compounds and the RI value, and the HQSAR model is capable of modeling this relationship. Second, the ensemble HQSAR models were established using the four individual HQSAR models with the highest accuracy as the sub-models through arithmetic averaging. The ensemble HQSAR models were validated by external test set validation and LOO-CV. The $q_{\mathrm{cv}}^{2}$ , CCC, $q_{\mathrm{ext}}^{2}$ , $Q_{F2}^{2}$ , and $Q_{F3}^{2}$ for predicting the RI values of the DB-210 and HP-Innowax stationary phases were 0.927 and 0.919, 0.956 and 0.979, 0.929 and 0.963, 0.927 and 0.958, and 0.935 and 0.963, respectively. Compared to the individual HQSAR models, the established ensemble HQSAR models show better robustness and accuracy, thus establishing that ensemble modeling is an effective approach. The combination of HQSAR and the ensemble modeling method is a practicable and promising method for studying and predicting the RI values of aldehydes and ketones.

全息定量构效关系(hologram quantitative structure-activity relationship, HQSAR)是一种以分子的亚结构片段(即分子全息)为结构描述符的定量构效关系(quantitative structure-activity relationship, QSAR)方法,具有建模简便快速、预测准确度高的特点,已广泛应用于化学、生物学、医学等众多领域^[1,2,3,4,5,6]^。HQSAR方法通常是建立分子全息描述符与样品性质之间的个体偏最小二乘(partial least squares, PLS)回归模型。但个体回归模型容易欠拟合和过拟合^[7]^,为了获得更准确可靠的回归模型,可以训练多个个体模型,通过一定的结合策略,形成一个综合了多个个体模型的集成模型。这种综合多个个体模型的方法称为集成建模(ensemble modeling)。集成建模方法可以克服使用单一个体模型的缺陷,提高模型的预测能力^[8,9,10]^,已经成功应用于QSAR建模、光谱分析、机器学习和人工智能等领域^[11,12,13,14]^。有必要研究能否应用集成建模方法提高HQSAR模型的预测能力。

色谱保留指数(retention index, RI)是色谱分析中的重要参数^[15,16]^。醛酮化合物种类众多,实验测定其RI值的时间和经济成本高,不同化合物在不同极性固定相上具有不同的保留行为,有必要建立不同极性固定相上醛酮化合物的RI值QSAR模型^[17,18,19]^。DB-210和HP-Innowax固定相具有强极性和高的使用温度上限,可用于醇类、硫醚类、脂类和醛酮类化合物色谱保留指数的测定。因此,本研究应用集成建模结合HQSAR方法研究了醛酮化合物在DB-210和HP-Innowax固定相上的色谱保留指数QSAR模型。

## 1 实验与方法

### 1.1 数据集

用于研究的34种醛酮化合物(如表1所示)在2种不同极性固定相DB-210和HP-Innowax上的RI实验值引自文献^[20]^。将34种化合物随机分为两组:第Ⅰ组(Group Ⅰ)包括26种化合物;第Ⅱ组(Group Ⅱ)包括8种化合物。

**表1:** 34种醛酮化合物在两种色谱柱上的保留指数实验值^[20]^与预测值

Group	Compound	RI of DB-210							RI of HP-Innowax										
		Exp	Pred			RE/%			Pred	Pred			RE/%						
Ind	Ens	Ind	Ens	Ind	Ens		Ind	Ens
Ⅰ	acetone	792.9	764.8	792.9		-3.54	0.00		835.0						877.3	879.1		5.07	5.28
	2-butanone	882.1	869.9	872.6		-1.38	-1.08		919.8						917.5	915.6		-0.25	-0.46
	3-methyl-2-butanone	943.3	943.8	934.1		0.05	-0.98		949.4						951.8	948.4		0.25	-0.11
	3-pentanone	960.8	970.2	959.3		0.98	-0.16		996.9						972.3	968.0		-2.47	-2.90
	3,3-dimethyl-2-butanone	992.0	991.6	982.8		-0.04	-0.93		968.5						904.8	893.7		-6.58	-7.72
	4-methyl-2-pentanone	1027.1	1004.2	995.6		-2.23	-3.07		1025.2						1041.4	1033.6		1.58	0.82
	2,4-dimethyl-3-pentanone	1038.5	1084.2	1077.8		4.40	3.78		1014.8						971.1	1010.7		-4.31	-0.40
	2-hexanone	1081.5	1079.0	1075.6		-0.23	-0.55		1097.2						1100.2	1085.6		0.27	-1.06
	4-heptanone	1134.5	1126.9	1122.3		-0.67	-1.08		1139.4						1148.0	1156.6		0.75	1.51
	5-methyl-2-hexanone	1161.3	1122.9	1118.4		-3.31	-3.69		1156.1						1139.5	1148.8		-1.44	-0.63
	2-heptanone	1184.3	1186.6	1185.7		0.19	0.12		1195.8						1211.3	1183.5		1.30	-1.03
	2-methyl-3-heptanone	1192.4	1173.2	1164.1		-1.61	-2.37		1178.7						1201.5	1210.8		1.93	2.72
	5-methyl-3-heptanone	1026.9	1172.2	1184.1		14.15	15.31		1200.1						1166.2	1190.4		-2.82	-0.81
	3-octanone	1255.5	1252.3	1254.0		-0.25	-0.12		1265.5						1262.5	1257.1		-0.24	-0.66
	5-nonanone	1342.6	1310.0	1325.4		-2.43	-1.28		1334.1						1345.1	1361.4		0.82	2.05
	acrolein	743.7	770.1	776.1		3.55	4.36		867.0						853.0	855.0		-1.61	-1.38
	isobutanal	803.7	839.9	839.3		4.50	4.43		830.4						836.8	877.7		0.77	5.70
	butanal	843.1	860.5	859.3		2.06	1.92		894.8						937.4	927.7		4.76	3.68
	isovaleraldehyde	912.8	919.1	908.1		0.69	-0.51		936.0						951.3	963.9		1.63	2.98
	2-methylbutanal	913.3	893.9	890.2		-2.12	-2.53		931.2						897.2	920.6		-3.65	-1.14
	valeraldehyde	953.8	966.8	959.3		1.36	0.58		998.1						1037.6	1007.9		3.96	0.98
	2-butenal	967.2	897.7	912.7		-7.19	-5.63		1061.5						953.8	955.2		-10.15	-10.01
	2-ethylbutyraldehyde	1009.6	1000.9	989.8		-0.86	-1.96		1018.0						1146.8	1112.5		12.65	9.28
	hexanal	1059.3	1113.6	1133.3		5.13	6.99		1098.3						1135.8	1156.0		3.41	5.25
	heptanal	1162.7	1149.4	1148.4		-1.14	-1.23		1199.6						1207.7	1176.9		0.68	-1.89
	octanal	1265.4	1240.9	1238.5		-1.94	-2.13		1298.8						1265.8	1249.2		-2.54	-3.82
Ⅱ	2-pentanone	973.9	979.3	978.7		0.55	0.49		996.2						1011.0	993.0		1.49	-0.32
	3-methyl-2-pentanone	1036.1	1023.7	1021.1		-1.20	-1.45		1033.9						1021.6	1008.8		-1.19	-2.43
	3-hexanone	1048.4	1043.9	1042.3		-0.43	-0.58		1068.0						1055.9	1058.4		-1.13	-0.90
	3-heptanone	1153.6	1132.8	1139.2		-1.80	-1.25		1167.2						1154.8	1158.0		-1.06	-0.79
	propanal	739.4	760.0	764.3		2.79	3.37		808.8						855.9	856.7		5.82	5.92
	trimethylacetaldehyde	841.6	876.4	900.8		4.13	7.03		822.6						779.7	840.5		-5.22	2.18
	3,3-dimethylbutanal	978.4	943.3	938.1		-3.59	-4.12		968.6						885.9	942.6		-8.54	-2.68
	2-ethylhexanal	1205.4	1109.1	1129.7		-7.99	-6.28		1197.8						1189.8	1240.2		-0.67	3.54

Exp: experimental; Pred: predicted; Ind: individual; Ens: ensemble; RE: relative error.

综合使用均方根误差(root mean square error, RMSE)、平均绝对百分比误差(mean absolute percentage error, MAPE)、交叉验证相关系数(squared correlation coefficient of cross validation, $q_{\mathrm{cv}}^{2}$ )、外部验证相关系数(squared correlation coefficient of external validation, $q_{\mathrm{ext}}^{2}$ )、一致性相关系数(concordance correlation coefficient, CCC)、预测平方相关系数F2(predictive squared correlation coefficient, $Q_{F2}^{2}$ )和F3( $Q_{F3}^{2}$ )^[21]^评估模型的预测能力。

$q_{\mathrm{cv}}^{2}$ 和 $q_{\mathrm{ext}}^{2}$ 定义如式(1)所示:


(1a) $q_{\mathrm{CV}}^{2}=1-\frac{\sum_{i=1}^{n_{\mathrm{TR}}}\left(\hat{y}_{i}-y_{i}\right)^{2}}{\sum_{i=1}^{n_{\mathrm{TR}}}\left(y_{i}-\bar{y}_{\mathrm{TR}}\right)^{2}}$

(1b) $q_{\mathrm{ext}}^{2}=1-\frac{\sum_{i=1}^{n_{\mathrm{EXT}}}\left(\hat{y}_{i}-y_{i}\right)^{2}}{\sum_{i=1}^{n_{\mathrm{EXT}}}\left(y_{i}-\bar{y}_{\mathrm{TR}}\right)^{2}}$

CCC、 $Q_{F2}^{2}$ 和 $Q_{F3}^{2}$ 的定义如式(2)和式(3)所示:


(2) $\mathrm{CCC}=\frac{2 \sum_{i=1}^{n_{\mathrm{EXT}}}\left(y_{i}-\bar{y}\right)\left(\hat{y}_{i}-\overline{\hat{y}}\right)}{\sum_{i=1}^{n_{\mathrm{EXT}}}\left(y_{i}-\bar{y}\right)^{2}+\sum_{i=1}^{n_{\mathrm{EXT}}}\left(\hat{y}_{i}-\overline{\hat{y}}\right)^{2}+n_{\mathrm{EXT}}(\bar{y}-\overline{\hat{y}})^{2}}$

(3a) $Q_{\mathrm{F} 2}^{2}=1-\frac{\sum_{i=1}^{n_{\mathrm{EXT}}}\left(\hat{y}_{i}-y_{i}\right)^{2}}{\sum_{i=1}^{n_{\mathrm{EXT}}}\left(y_{i}-\bar{y}_{\mathrm{EXT}}\right)^{2}}$

(3b) $Q_{\mathrm{F} 3}^{2}=1-\frac{\left[\sum_{i=1}^{n_{\mathrm{EXT}}}\left(\hat{y}_{i}-y_{i}\right)^{2}\right] / n_{\mathrm{EXT}}}{\left[\sum_{i=1}^{n_{\mathrm{TR}}}\left(y_{i}-\bar{y}_{i}\right)^{2}\right] / n_{\mathrm{TR}}}=1-\frac{\mathrm{PRESS} / n_{\mathrm{EXT}}}{\mathrm{TSS} / n_{\mathrm{TR}}}$

式(1)、式(2)和式(3)中:*y_i_*表示各样本的实验值,$\hat{y}_{i}$表示各样本的预测值, $\bar{y}$ 表示全部样本实验值的平均值,$\overline{\hat{y}}$表示全部样本预测值的平均值,*n*_EXT_和*n*_TR_分别表示外部测试集和训练集样本数,PRESS是预测误差平方和,TSS是全部样本的预测误差平方和。一般认为,QSAR模型的CCC应大于0.85, $Q_{F2}^{2}$ 和 $Q_{F3}^{2}$ 应大于0.60, $q_{\mathrm{cv}}^{2}$ 和 $q_{\mathrm{ext}}^{2}$ 应大于0.50^[22,23]^。

### 1.2 实验过程

1.2.1 分子模型构建及构象优化

使用SYBYL-X 2.0软件(Tripos公司,美国)构建34种醛酮化合物的计算机分子模型。采用Tripos分子力场进行构象优化,加载Gasteiger-Huckel电荷,能量收敛标准设定为2.09 J/(mol·nm) (0.005 kcal/(mol·Å)),迭代次数1000次,其余参数均采用SYBYL程序的默认值,得到各分子的最低能量构象用于HQSAR建模。

1.2.2 分子全息产生及HQSAR模型构建

HQSAR方法将分子结构划分为包含所有可能结构(线性、分支、环状、搭接或重叠)的分子片段(molecular fragments),再将分子片段进行编码使之转化为分子全息(molecular hologram)。分子结构片段的特征主要由片段特性(fragment distinction, FD)和片段尺寸(fragment size, FS)两个参数规定。FD参数可以选择的值包括原子(atoms, A)、化学键(bonds, B)、连接(connections, C)、氢原子(hydrogen atoms, H)、手性(chirality, Ch)和供体/受体原子(donor/acceptor atoms, DA)。A可以区分不同类型的原子;B可以识别原子形成化学键之间的差异;C可以描述片段内原子的杂化状态;Ch可以描述片段中原子和化学键的立体化学信息;H可以描述分子片段的氢键供体或受体情况^[24]^。FS参数值包括最小原子数(*M*)和最大原子数(*N*)。通常*M*的取值最小从2开始;*N*的取值须大于*M*,最大值一般为12且不超过分子的总原子数。通过环丰度检验算法(cyclic redundancy check, CRC)计算每个结构特征碎片出现的频率,将各个分子碎片映射为0~231的伪随机整数,使得每个分子可被表示为一定长度的整数串,进而采用Hashing算法将它们转换为具有相同长度的整数串,即为分子全息^[25]^。在SYBYL-X 2.0的HQSAR模块中,分子全息长度(hologram lengths, HL)从系统默认的6个值:97、151、199、257、307和353中进行选择。用PLS方法建立化合物分子全息与性质之间的HQSAR回归模型。通过调整FD、FS及全息长度等参数来优化模型^[26]^。

1.2.3 集成模型构建

集成模型的构建通常由两个步骤组成:(1)建立一系列多样化的个体模型(子模型); (2)采用适当的集成规则,对各子模型的预测结果进行集成,得到集成(共识)结果。主要思路是通过多个模型的集成,个体模型的误差可被其他多个个体模型所补偿从而使得集成模型的整体性能优于个体模型。要获得好的集成模型,各子模型应该具有一定的准确度,且子模型应该足够多样化。因此,本文采用由不同参数建立的个体HQSAR模型作为子模型(*h*_1_, *h*_2_, *h*_3_, …, *h*_T_)建立集成HQSAR模型,以各子模型预测值的算术平均值作为集成模型的预测结果,如式(4)所示:


(4) *H*(*x*)*=*$\frac{1}{T}\sum_{1}^{T}$*h_i_*(*x*)

式(4)中:*h_i_*(*x*)表示各子模型的预测值,*T*表示子模型的个数。

## 2 结果与讨论

### 2.1 个体HQSAR模型

以第Ⅰ组为训练集,优化FD和FS参数。首先,使用默认的FS参数值4~7,选取不同FD参数建立20个HQSAR模型。表2所列为其中6个最佳模型的主要统计量。由表2可知,在固定相DB-210和HP-Innowax上,FD参数值为“DA”时可以得到最优模型,其 $q_{\mathrm{cv}}^{2}$ 分别为0.913和0.909。其次,设置FD参数值为“DA”,选用不同的FS参数建立40个HQSAR模型以选择合理的FS参数。表3所列为其中4个最优模型的主要统计量。由表3可知,在固定相DB-210上,FS参数值为1~9时可得到最优模型,在固定相HP-Innowax上,FS参数值为4~7时可得到最优模型,其 $q_{\mathrm{cv}}^{2}$ 分别为0.935和0.909, *r*^2^分别为0.990和0.978。由上述研究可知,对固定相DB-210,建立最佳个体HQSAR模型的条件为:FD为“DA”, FS为1~9, HL为151,主成分数(principal components, PCs)为5;对固定相HP-Innowax,建立最佳个体HQSAR模型的条件为:FD为“DA”, FS为4~7, HL为151, PCs为6。

**表2:** 不同片段特性的全息定量构效关系(HQSAR)模型统计参数

Fragment distinction	DB-210					HP-Innowax					
	$q_{\mathrm{cv}}^{2}$	SEE	HL	PCs		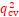		SEE	HL	PCs	
B	0.801	37.357	257	4		0.879		27.872	199	4	
DA	0.913	21.6	97	5		0.909		24.377	151	6	
A+DA	0.852	26.059	353	5		0.893		33.318	353	3	
B+DA	0.804	34.375	257	4		0.859		47.967	151	2	
A+C+DA	0.856	27.91	97	5		0.862		26.596	97	5	
A+B+H+DA	0.821	24.813	353	5		0.883		26.959	257	4	

$q_{\mathrm{cv}}^{2}$: squared correlation coefficient of cross validation; SEE: standard error of estimate; HL: hologram lengths; PCs: principal components.

**表3:** 不同片段尺寸的HQSAR模型统计参数

DB-210							HP-Innowax						
Fragment size	$q_{\mathrm{cv}}^{2}$	*r* ^2^	SEE	HL	PCs		Fragment size	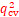		*r* ^2^	SEE	HL	PCs
1月9日	0.935	0.99	17.124	151	5		4月7日	0.909		0.978	24.377	151	6
3月10日	0.921	0.99	17.71	151	5		1月9日	0.908		0.937	37.685	97	2
4月7日	0.913	0.984	21.6	97	5		3月10日	0.908		0.972	26.202	151	4
2月5日	0.897	0.955	36.017	151	4		3月6日	0.894		0.962	30.662	151	4

用外部测试集验证和留一交叉验证评估HQSAR模型的预测能力。外部测试集验证以第Ⅰ组作为训练集,采用上述最佳建模条件建立不同固定相上的个体HQSAR模型,预测第Ⅱ组8个化合物的RI值,结果如表1及表4所示。用第Ⅰ组完成留一交叉验证,即共进行26次预测,每次选定一个样本作为测试集,以其余25个样本作为训练集,仍然采用上述最佳建模条件建立模型,依次预测26个醛酮化合物的RI值,结果如表1及表4所示。外部测试集验证和留一交叉验证结果表明醛酮化合物的分子全息描述符与RI值之间存在一定的定量关系,建立的HQSAR模型准确可靠。与在固定相HP-Innowax上建立的模型相比,在固定相DB-210上所建立的最佳个体HQSAR模型准确度更高。

**表4:** 个体HQSAR模型与集成HQSAR模型的统计参数

Parameter	DB-210			HP-Innowax	
	Individual model	Ensemble model		Individual model	Ensemble model
RMSE	39.123	40.417		41.451	37.553
MAPE	2.60%	2.69%		2.97%	2.74%
CCC	0.953	0.956		0.960	0.979
$q_{\mathrm{ext}}^{2}$	0.925	0.929		0.927	0.963
$Q_{F2}^{2}$	0.922	0.927		0.918	0.958
$Q_{F3}^{2}$	0.931	0.935		0.927	0.963
$q_{\mathrm{cv}}^{2}$	0.935	0.927		0.909	0.919

RMSE: root mean square error; MAPE: mean absolute percentage error; CCC: concordance correlation coefficient; $q_{\mathrm{ext}}^{2}$: squared correlation coefficient of external validation; $Q_{F2}^{2}$, $Q_{F3}^{2}$: predictive squared correlation coefficient F2, F3.

### 2.2 集成HQSAR模型

在选用不同参数建立的40个HQSAR模型中,以表3所列4个准确度最高的个体HQSAR模型作为子模型通过算术平均建立集成HQSAR模型,即集成模型对各化合物的RI预测值是这4个模型预测值的平均值。由表3可知,这些子模型预测准确度较高且有一定的差异,符合集成建模要求。用外部测试集验证和留一交叉验证评估集成模型的预测能力。在外部测试集验证中,用建立的集成模型预测第Ⅱ组中8个化合物的RI值,结果见表1。用第Ⅰ组的26个样本完成留一交叉验证,结果见表1及表4。外部测试集验证和留一交叉验证结果表明建立的集成模型准确可靠。固定相DB-210的集成HQSAR模型与个体HQSAR模型预测准确度非常接近。这主要由于此个体HQSAR模型的预测准确度相对较高,不易通过改变建模方法提高模型的准确度,因此集成建模并不能显著改进模型的预测能力。对固定相HP-Innowax,由表1可得,对于第Ⅰ组中相对误差最大的化合物2-乙基丁醛,通过集成建模,相对误差由12.65%降低到9.28%;对于第Ⅱ组中相对误差最大的化合物3,3-二甲基丁醛,通过集成建模,相对误差由-8.54%降低到-2.68%。由表4可以看出,集成模型的所有预测评估参数均优于个体HQSAR模型,相对于个体模型的评估参数,集成模型的RMSE和MAPE值分别降低了3.898和0.23%,与原RMSE和MAPE值相比降低了9.40%和7.74%; CCC、 $q_{\mathrm{ext}}^{2}$ 、 $Q_{F2}^{2}$ 、 $Q_{F3}^{2}$ 和 $q_{\mathrm{cv}}^{2}$ 值分别升高了0.019、0.036、0.04、0.036和0.01,与原CCC、 $q_{\mathrm{ext}}^{2}$ 、 $Q_{F2}^{2}$ 、 $Q_{F3}^{2}$ 和 $q_{\mathrm{cv}}^{2}$ 值相比升高了1.98%、3.88%、4.36%、3.88%和1.10%。显然通过集成建模可以显著提高个体HQSAR模型中一些预测误差较大化合物的预测结果,从而提高HQSAR模型的稳健性和准确度。

## 3 结论

对34种醛酮化合物的HQSAR集成建模研究证明,醛酮化合物的分子结构与RI值之间存在定量关系,可以建立醛酮化合物RI值的个体HQSAR模型。应用集成建模方法对个体HQSAR模型进行集成,则可以提高对RI值的预测准确度。这说明集成建模是一种提高HQSAR模型稳健性和准确度的有效方法,HQSAR方法与集成建模方法相结合可以用于研究和预测醛酮化合物的RI值。
